# Mechanisms of the impact of exercise intervention on college students' mental health: a longitudinal experimental study using swimming as an example

**DOI:** 10.3389/fpsyg.2025.1535214

**Published:** 2025-06-16

**Authors:** Wen Wang, Linling Yu, Le Huang, Xing Gao

**Affiliations:** ^1^School of Physical Education, Hunan University of Science and Technology, Xiangtan, Hunan, China; ^2^Taekwondo Sports, Woosuk University, Jeonju, Republic of Korea; ^3^College of Physical Education, Woosuk University, Jeonju, Republic of Korea; ^4^College of Education, Infrastructure University Kuala Lumpur, Kuala Lumpur, Malaysia

**Keywords:** college student mental health, swimming, emotional stability, social adaptation, academic stress

## Abstract

**Introduction:**

With the growing prevalence of mental health issues among college students, there is a pressing need for effective and accessible intervention strategies. This study explores the potential of swimming as a structured intervention to improve students' emotional well-being, social adaptation, and academic stress management.

**Methods:**

A 15-week swimming intervention program was implemented among 44 college students, who were randomly assigned to either an experimental group (swimming intervention) or a control group (no intervention). Psychological assessments were conducted before and after the intervention to evaluate changes in emotional state, social functioning, and academic stress.

**Results:**

Students in the experimental group showed significant improvements in emotional stability (3.85 ± 0.78 vs. 3.52 ± 0.80, *P* = 0.01) and relaxation (3.82 ± 0.75 vs. 3.25 ± 0.61, *P* = 0.02). Positive changes were also observed in social adaptation, including interpersonal harmony (4.14 ± 0.73, *P* = 0.03) and perceived social support (4.21 ± 0.75, *P* = 0.04). Additionally, academic composure (3.57 ± 0.82 vs. 3.05 ± 0.83, *P* = 0.02) and goal management ability (3.73 ± 0.78, *P* = 0.04) significantly improved.

**Discussion:**

The findings demonstrate that a structured swimming program can be an effective intervention for enhancing mental health among college students. It fosters emotional regulation, strengthens social ties, and improves coping strategies for academic stress. Further research is recommended to assess the long-term benefits and generalizability across broader populations.

## 1 Introduction

Mental health issues among college students have garnered global attention, especially in the context of modern higher education, where rising stress and uncertainty contribute to increasing rates of depression, anxiety, and other mental health concerns each year. These issues not only impact students' academic performance and interpersonal relationships but also pose long-term risks to their future quality of life and career prospects (Moeller et al., 2020; Syifa et al., 2023). Traditional mental health interventions, such as psychotherapy and pharmacotherapy, have demonstrated efficacy but also have certain limitations, including high costs, low adherence, and potential side effects (Son et al., 2020; Xiao et al., 2022). Consequently, identifying effective methods to enhance college students' mental health has become a crucial focus for academia and educational management.

Exercise intervention, a natural, non-pharmacological approach, has gained increasing attention in recent years for its potential to improve mental health. Exercise not only regulates mood by promoting the secretion of neurotransmitters like dopamine and serotonin, but it also boosts self-esteem, enhances self-efficacy, and improves sleep quality (Li and Chen, 2023). Moreover, the social aspects of exercise provide students with additional sources of emotional support, which can effectively alleviate feelings of loneliness and social anxiety (Park et al., 2023). Existing research has confirmed the positive effects of various forms of exercise interventions on mental health. In particular, moderate- to high-intensity aerobic activities such as running, cycling, and strength training have been widely recognized for their efficacy in reducing depression and anxiety while enhancing psychological resilience (Borrega-Mouquinho et al., 2021; Costa et al., 2022). Some longitudinal studies indicate that sustained exercise interventions not only aid in short-term mood improvement but also yield significant long-term benefits for emotional stability, social adaptability, and quality of life (Campo et al., 2020; Dowla et al., 2022). While much of the research has concentrated on the positive effects of land-based aerobic and strength exercises on mental health, there remains a notable gap in exploring aquatic exercises—particularly swimming, a full-body endurance activity involving major muscle groups—as a potential mental health intervention for college students. Swimming, as a unique aquatic exercise, possesses psychological regulation mechanisms that are unparalleled by other forms of physical activity (Tang et al., 2022). Firstly, the buoyancy of water alleviates joint pressure, making swimming particularly suitable for individuals with physical discomfort or sports injuries. This characteristic allows swimming to serve as a low-impact, full-body exercise that can be sustained over long periods without causing excessive fatigue. Secondly, the hydrostatic pressure effect effectively promotes blood circulation and reduces physical tension, thereby aiding in emotional regulation. This unique attribute provides distinct advantages in alleviating anxiety and tension while enhancing emotional stability. Lastly, the coordination of breathing rhythm and bodily movements required in swimming strengthens self-control and concentration, helping to mitigate emotional fluctuations and improve emotional stability. To address these gaps, this study employs a longitudinal comparative experiment to systematically evaluate the long-term effects of swimming on college students' mental health across three dimensions: emotional state, social adaptation, and academic stress. The findings reveal the mechanisms through which swimming impacts mental health, providing theoretical support for higher education institutions to formulate more practical mental health intervention programs, as well as offering new insights for the development of public health policies.

The structure of this paper is as follows: The first section, the introduction, outlines the study background, problems, and objectives. The second section, the literature review, examines existing research on the application of exercise interventions in mental health. The third section describes the study design, including the selection of participants and experimental framework. The fourth section presents and analyzes the results of swimming's impact on college students' mental health. The fifth section, the discussion, summarizes the study findings, compares them with other research, highlights contributions and limitations, and provides recommendations. The final section, the conclusion, summarizes the study outcomes and suggests directions for future research.

## 2 Literature review

In today's fast-paced social environment, mental health has emerged as a critical global public health issue. Mental health challenges, such as depression, anxiety, and stress, not only affect individuals' daily lives but also have profound and lasting impacts on both physical and mental wellbeing. Regular exercise intervention has demonstrated significant positive effects on mental health; by promoting the release of endorphins and other mood-enhancing neurotransmitters, exercise can directly improve mood while helping individuals foster a more positive self-image and establish stronger social connections (Tikac et al., 2021; Mahindru et al., 2023). Moreover, exercise type, intensity, and frequency can substantially influence the mental health benefits achieved (Zhu et al., 2020; Ji et al., 2022). As exercise science and psychology increasingly intersect, exploring the specific mechanisms of different exercise interventions on mental health holds valuable research and practical implications.

As a non-pharmacological treatment, exercise intervention is considered effective for improving mental health. Pascoe et al. (2020), in a systematic review, indicated that moderate- to high-intensity exercise interventions demonstrate significant antidepressant and anxiolytic effects in both adolescent and adult populations. Althumiri et al. (2020) found that among Saudi Arabian adults who met the World Health Organization's recommendations for moderate-intensity physical activity, depressive, and anxiety symptoms were significantly reduced, thus affirming the mental health benefits of moderate exercise. A cross-sectional study by Sugano et al. (2022) in Japan also showed that any level of exercise positively impacted workers' self-rated health scores, underscoring that exercise interventions can have beneficial mental health effects in occupational settings as well. Overall, substantial research supports the positive association between exercise interventions and mental health, particularly the enhanced benefits of moderate- to high-intensity activities. Wang et al. (2023) further observed that, during the COVID-19 pandemic, moderate-intensity exercise performed 3–5 times per week for 30–40 min significantly reduced symptoms of anxiety and depression, while also improving participants' quality of life. Additionally, the intensity and frequency of exercise play a key role in achieving mental health benefits. An evaluation of high-intensity interval training (HIIT) by Martland et al. (2020) revealed that HIIT not only improved participants' cardiovascular health but also significantly reduced depressive symptoms, indicating the potential mental health benefits of high-intensity exercise, although such regimens may require higher endurance and adherence. Hallgren et al. (2020) found that engaging in moderate-intensity exercise 3–5 times weekly effectively reduced symptoms of anxiety and depression, though excessive sedentary time could undermine these benefits. Wassenaar et al. (2021) reported that, although HIIT enhanced physical fitness among adolescents, it did not significantly improve mental health, suggesting that individual tolerance for varying exercise intensities affects the psychological benefits of exercise interventions. Research by Imai et al. (2024) demonstrated that more than 50 min of moderate- to high-intensity exercise per week significantly alleviated anxiety and depression in breast cancer patients, also improving body image and quality of life.

Among various mental health issues, exercise intervention has shown notable efficacy. For instance, Chen et al. (2020) found that exercise interventions for cancer patients not only reduced depressive symptoms but also significantly improved quality of life, thus providing scientific support for the use of exercise as a mental health intervention in cancer care. In a study of adolescents from low socioeconomic backgrounds, Poon (2021) noted that both aerobic and high-intensity interval training were effective in reducing anxiety and depression symptoms, presenting new pathways for supporting mental health in special populations. Efthymiou et al. (2021) concluded that exercise interventions positively impacted the mental health of overweight and obese individuals, especially by enhancing quality of life and self-efficacy. Additionally, Burn et al. (2021), in a study on working adults, observed that moderate exercise improved mental health in the workplace, particularly in high-stress environments.

While the positive effects of exercise interventions on mental health are widely supported, many existing studies use cross-sectional designs and lack longitudinal experimental data, limiting our understanding of the long-term effects of these interventions. Furthermore, most exercise intervention research has focused on short-term, land-based activities. To address these issues, this study employs a longitudinal experimental design to investigate the long-term impact mechanisms of swimming on college students' mental health, with a particular focus on the comprehensive effects and mechanisms across different mental health indicators.

## 3 Research design

### 3.1 Participants

The study participants were college students aged 18–24, all of whom were physically healthy and exhibited stable mental states. To ensure scientific rigor, none of the participants had received prior professional swimming training, allowing for a clearer assessment of the effects of the swimming intervention. The study initially distributed 200 online questionnaires to students meeting the eligibility criteria, with a total of 182 valid responses returned, achieving a 91% response rate. After screening for full compliance with the study criteria, 44 students were selected as subjects and randomly assigned to either the experimental or control group, with balanced numbers in each group to ensure comparability of the intervention effects. After selecting 44 eligible participants, a computer-generated simple randomization method was employed to ensure balance and randomness between the experimental and control groups. Specifically, each participant was assigned a unique identification number and randomly allocated using Python's numpy.random.choice function, ensuring an even distribution between the experimental group (*n* = 22) and the control group (*n* = 22). To minimize selection bias, researchers remained blinded to the allocation process before group assignment, thereby improving the fairness and reproducibility of the study. This approach further enhances the transparency of the study design and provides a solid foundation for evaluating the effectiveness of the intervention.

The experiment was conducted over a period of 15 weeks, from April to July 2023. Before the intervention began, baseline data on participants, including height, weight, and exercise habits, were collected via questionnaire. Psychological health assessments were administered to all participants both before and after the intervention. Additionally, all participants signed informed consent forms to ensure voluntary participation, and data were processed anonymously to uphold ethical standards, providing a scientifically sound basis for evaluating the intervention's effects.

### 3.2 Mental health assessment

As shown in Table 1, the mental health assessment questionnaire used in this study was designed to measure three primary dimensions: emotional state, social adaptation, and academic stress, each encompassing nine specific indicators. The questionnaire included 27 items, with three sub-items for each indicator, and used a five-point Likert scale for scoring. Participants rated each item based on their individual experiences, with final scores calculated as averages to provide a comprehensive analysis of mental health status. This structured questionnaire provided a foundational dataset for evaluating the intervention effects, facilitating a thorough and accurate reflection of college students' mental health levels. The detailed questionnaire is attached at the end of the document.

**Table 1:** Dimensions of the mental health assessment.

**Dimension**	**Indicator**	**Description**
Emotional state	Emotional relaxation	Reflects the degree of calmness and relaxation in students' emotions during daily life.
	Positive emotion experience	Indicates the frequency with which students experience positive emotions such as happiness, confidence, and satisfaction.
	Emotional stability	Represents the stability of students' emotional fluctuations, indicating the persistence and consistency of emotions.
Social adaptation	Interpersonal harmony	Indicates the degree of harmony and satisfaction in students' interactions with classmates, friends, and family.
	Sense of Social support	Reflects the strength of emotional and practical support that students receive from family, friends, and society.
	Communication confidence	Represents students' confidence in expressing themselves and communicating effectively in social settings.
Academic stress	Academic composure	Reflects students' sense of ease when facing academic tasks and course requirements.
	Sense of academic achievement	Represents students' satisfaction with their academic performance and progress.
	Goal management ability	Reflects students' ability to set academic goals, follow plans, and achieve those goals.

### 3.3 Experimental method

#### 3.3.1 Experimental environment and conditions

To ensure scientific rigor, consistency, and participant safety, the school swimming pool was selected as the intervention environment, with strict control of environmental conditions. The pool water temperature was maintained between 26 and 28°C to provide a comfortable training atmosphere, avoiding temperature fluctuations that might impact physical and mental wellbeing. The pool's humidity, ventilation, and water quality were all kept within standard limits to ensure proper air circulation, thereby minimizing environmental biases. Training sessions were scheduled at a consistent daily time to reduce the effects of biological rhythm and lifestyle stress fluctuations. Additionally, participants were instructed to maintain regular sleep patterns and avoid intense physical activity or sudden psychological stressors during the experiment to preserve the intervention's effect purity. Professional coaches or lab assistants provided continuous guidance throughout the training, ensuring that students adhered strictly to standardized training routines and paces. To further enhance safety, heart rate, and emotional state were recorded before and after each session, allowing real-time monitoring of participants' physical and mental status and enabling prompt support when needed. These rigorous controls and safety protocols created a scientific, stable, and safe intervention environment for the experimental group, facilitating an accurate evaluation of the swimming intervention's impact on mental health.

#### 3.3.2 Swimming training program

As shown in Table 2, the swimming intervention program lasted 15 weeks, with training frequency and duration gradually increasing to provide structured swimming skills training for the experimental group. The intervention was divided into multiple stages, progressively moving from basic water acclimation exercises to endurance and skill enhancement. This phased approach allowed participants to gradually adapt to the physical demands of the training, supporting the goal of improving mental health.

**Table 2:** Swimming intervention program.

**Phase**	**Intervention period**	**Frequency**	**Duration (per session)**	**Training content**
Adaptation	Weeks 1–2	2 times/week	20 min	Water acclimation, breath-holding, floating, and basic kicking exercises to help participants overcome tension in the water.
Foundation	Weeks 3–4	2 times/week	30 min	Learning freestyle kicking and breathing techniques, gradually adapting to water breathing rhythm, continuing floating practice.
Improvement	Weeks 5–6	3 times/week	30 min	Practicing complete freestyle kicking movements, introducing basic arm strokes to develop preliminary movement coordination.
Skills advancement	Weeks 7–9	3 times/week	40 min	Practicing continuous freestyle movements, strengthening breathing and movement coordination, with introductory breaststroke kicks.
consolidation	Weeks 10–12	3 times/week	50 min	Alternating freestyle and breaststroke practice, gradually increasing endurance and distance to establish foundational swimming skills.
Integration	Weeks 13–15	3 times/week	60 min	Continuous freestyle and breaststroke practice, with the addition of basic speed and endurance training to consolidate skills and enhance performance.

## 4 Results and analysis

### 4.1 Comparison of basic information

To ensure the scientific rigor and validity of the experiment, a statistical analysis of baseline characteristics was conducted for both the experimental and control groups. The primary indicators included height, weight, and exercise habits (further broken down into exercise frequency and duration). A *t*-test was used to analyze differences between the two groups for these indicators. Table 3 presents the results of the comparison. As shown in Table 3, the mean height of the experimental and control groups was 170.45 ± 5.32 cm and 169.82 ± 5.27 cm, respectively; the mean weight was 65.30 ± 7.11 kg for the experimental group and 65.18 ± 6.98 kg for the control group. Exercise frequency averaged 1.55 ± 0.53 times per week for the experimental group and 1.51 ± 0.43 times for the control group, while exercise duration was 29.81 ± 5.67 minutes for the experimental group and 30.10 ± 5.48 min for the control group. The *t*-test results indicate that the *P*-values for height, weight, exercise frequency, and exercise duration were all >0.05, signifying no significant differences in these baseline characteristics between the experimental and control groups. This lack of baseline discrepancies ensures the scientific validity and comparability of the intervention effects.

**Table 3:** Comparison of basic information between experimental and control groups.

	**Height (cm)**	**Weight (kg)**	**Exercise frequency (times/week)**	**Exercise duration (min)**
Experimental group	170.45 ± 5.32	65.30 ± 7.11	1.55 ± 0.53	29.81± 5.67
Control group	169.82 ± 5.27	65.18 ± 6.98	1.51 ± 0.43	30.10 ± 5.48
*T*-values	0.15	0.18	0.37	0.21
*P*-values	0.88	0.86	0.93	0.81

### 4.2 Homogeneity test

Before the intervention, a homogeneity test was conducted on the emotional state, social adaptation, and academic stress dimensions for both the experimental and control groups. The specific results are shown in Table 4. As indicated in Table 4, the following findings were observed: In the emotional state dimension, the relaxation score for the experimental group was 3.26 ± 0.81, while it was 3.15 ± 0.81 for the control group, yielding a *P*-value of 0.67, suggesting no significant difference between the groups. The positive emotion experience score was 3.75 ± 0.74 in the experimental group and 3.63 ± 0.72 in the control group, with a *P*-value of 0.69, again indicating no significant difference. In terms of emotional stability, the scores were 3.52 ± 0.91 for the experimental group and 3.48 ± 0.92 for the control group, with a *P*-value of 0.72, showing no significant difference. For social adaptation, the experimental group scored slightly higher across all metrics, including interpersonal harmony (3.92 ± 0.77) compared to 3.87 ± 0.78 for the control group, sense of social support (4.03 ± 0.82 for the experimental group and 3.97 ± 0.80 for the control group), and communication confidence (3.86 ± 0.88 for the experimental group vs. 3.73 ± 0.88 for the control group). The *P*-values for interpersonal harmony, sense of social support, and communication confidence were 0.61, 0.54, and 0.51, respectively, indicating no significant differences in social adaptation metrics between the two groups. In the academic stress dimension, the academic composure score for the experimental group was 2.92 ± 0.81, compared to 2.85 ± 0.83 for the control group, with a *P*-value of 0.64, suggesting no significant difference. The sense of academic achievement scored 3.24 ± 0.82 in the experimental group and 3.13 ± 0.85 in the control group, with a *P*-value of 0.53, also showing no significant difference. Finally, the goal management ability score was 3.13 ± 0.87 for the experimental group and 3.07 ± 0.84 for the control group, yielding a *P*-value of 0.70, again indicating no significant difference.

**Table 4:** Homogeneity test results.

**Indicator**		**Group**		***T*-values**	***P*-values**
		**Experimental group**	**Control group**		
Emotional state	Emotional relaxation	3.26 ± 0.81	3.15 ± 0.81	0.42	0.67
	Positive emotion experience	3.75 ± 0.74	3.63 ± 0.72	0.88	0.69
	Emotional stability	3.52 ± 0.91	3.48 ± 0.92	0.36	0.72
Social adaptation	Interpersonal harmony	3.92 ± 0.77	3.87 ± 0.78	0.51	0.61
	Sense of social support	4.03 ± 0.82	3.97 ± 0.83	0.77	0.54
	Communication confidence	3.86 ± 0.88	3.73 ± 0.88	0.67	0.51
Academic stress	Academic composure	2.92 ± 0.81	2.86 ± 0.81	0.72	0.47
	Sense of academic achievement	3.24 ± 0.82	3.13 ± 0.85	0.63	0.53
	Goal management ability	3.13 ± 0.87	3.07 ± 0.93	0.39	0.70

In summary, the homogeneity test results in Table 4 demonstrate that there were no significant differences (all *P*-values > 0.05) between the experimental and control groups across the various indicators in the dimensions of emotional state, social adaptation, and academic stress. This confirms good baseline comparability between the two groups, providing a solid scientific foundation for the subsequent evaluation of the swimming intervention's effects.

### 4.3 Post-intervention psychological state assessment results

Table 5 presents the post-intervention assessment results for various psychological health indicators in both the experimental and control groups. After undergoing the swimming intervention, the experimental group showed significant improvements in emotional state, social adaptation, and academic stress, outperforming the control group in these aspects. In the emotional state dimension, the experimental group's score for emotional relaxation was 3.82 ± 0.75, while the control group scored 3.25 ± 0.61, with a *P*-value of 0.02. This indicates that the experimental group experienced significantly higher emotional relaxation, which may be attributed to the unique role of the water environment in promoting emotional relaxation. The buoyancy of water creates a sensation of floating, which helps to relax the body and reduce physical tension. In terms of positive emotional experience, the experimental group scored 4.07 ± 0.71, compared to 3.68 ± 0.74 for the control group (*P* = 0.01). This significant improvement can be explained by the release of endorphins during swimming, which are known as “happiness hormones,” leading students to experience greater emotional joy and satisfaction post-exercise. Regarding emotional stability, the experimental group's score was 3.85 ± 0.78, while the control group scored 3.52 ± 0.80 (*P* = 0.01), showing a significant improvement in emotional stability. The rhythmic breathing and coordination required in swimming likely helped students better manage emotional fluctuations, enhancing their ability to focus and regulate emotions, which contributed to greater emotional stability.

**Table 5:** Post-intervention psychological state assessment results.

**Indicator**		**Group**		***T*-values**	***P*-values**
		**Experimental group**	**Control group**		
Emotional state	Emotional relaxation	3.82 ± 0.75	3.25 ± 0.61	2.40	0.02
	Positive emotion experience	4.07 ± 0.71	3.68 ± 0.74	2.24	0.01
	Emotional stability	3.85 ± 0.78	3.52 ± 0.80	2.10	0.01
Social adaptation	Interpersonal harmony	4.14 ± 0.73	3.83 ± 0.76	2.05	0.03
	Sense of Social support	4.21 ± 0.75	3.90 ± 0.77	2.18	0.04
	Communication confidence	4.05 ± 0.78	3.78 ± 0.79	2.11	0.04
Academic stress	Academic composure	3.57 ± 0.82	3.05 ± 0.83	2.29	0.02
	Sense of academic achievement	3.84 ± 0.75	3.18 ± 0.77	2.15	0.04
	Goal management ability	3.73 ± 0.78	3.11 ± 0.80	2.09	0.04

In the social adaptation dimension, the experimental group showed notable improvements, with an interpersonal harmony score of 4.14 ± 0.73, compared to 3.83 ± 0.76 for the control group (*P* = 0.03). This suggests that the experimental group had significantly better social integration. The shared experience of swimming training, frequent interactions with coaches and peers, and the team-based environment provided ample opportunities for students to build better interpersonal relationships through communication, encouragement, and competition. For sense of social support, the experimental group scored 4.21 ± 0.75, while the control group scored 3.90 ± 0.77 (*P* = 0.04), indicating a significant increase in the experimental group's sense of social support. The mutual help and cooperation inherent in the swimming training environment strengthened the sense of support and trust among team members, fostering a strong sense of belonging and collective support that transferred to their daily lives. In terms of communication confidence, the experimental group scored 4.05 ± 0.78, compared to 3.78 ± 0.79 for the control group (*P* = 0.04), showing a significant improvement in communication confidence. This boost likely resulted from engaging in communication during swimming lessons, whether discussing techniques with coaches or coordinating training with peers, thus enhancing students' confidence in social interactions.

In the academic stress dimension, the experimental group also showed clear advantages. For academic composure, the experimental group scored 3.57 ± 0.82, while the control group scored 3.05 ± 0.83 (*P* = 0.02), indicating that the experimental group felt significantly more composed when facing academic stress. The physical and endurance training involved in swimming helped students strengthen their psychological resilience, enabling them to cope better with academic pressures. Regarding academic achievement sense, the experimental group's score was 3.84 ± 0.75, compared to 3.48 ± 0.77 for the control group (*P* = 0.04), showing a significant improvement. The sense of accomplishment in swimming—whether by reaching new speeds or completing longer distances—enhanced students' self-recognition. This achievement likely transferred to their academic lives, boosting their confidence in their academic progress and increasing their sense of academic accomplishment. Finally, in goal management ability, the experimental group scored 3.73 ± 0.78, while the control group scored 3.11 ± 0.80 (*P* = 0.04), showing a significant improvement. Swimming training, which often involves setting progressive goals, helped students develop a stronger sense of planning, self-discipline, and execution.

## 5 Discussion

This study employed a controlled experimental design to systematically assess the impact of swimming intervention on college students' mental health by comparing the performance differences between the experimental and control groups. The results indicated that after 15 weeks of swimming intervention, the experimental group exhibited significant improvements in emotional state, social adaptability, and academic stress management. This finding suggests that swimming, as a holistic form of exercise, not only enhances physical health but also effectively improves mental health. Compared to general psychological interventions, swimming provides a dual physical and psychological benefit, offering students a stable means of emotional regulation. This highlights its potential value in college mental health education.

Existing research generally supports the positive impact of exercise interventions on mental health in college students. However, this study presents innovation both in methodology and content, further expanding the application scenarios and making novel attempts in evaluation. Uniquely, this study adopted a phased, progressive swimming intervention program, with increasing training frequency and intensity, to help participants gradually adapt to physical exertion, leading to emotional regulation. In comparison, the study by Yue and Xiao (2022), although confirming the role of aerobic exercise in improving emotional stability, did not include a systematic variation in exercise intensity, resulting in less pronounced adaptation effects than those provided by a progressive swimming program. Furthermore, Doyenart et al. (2024) emphasized the positive impact of water environments on emotional regulation, but their study did not explore how specific exercises could leverage the characteristics of water environments for mental health interventions. Building on this theoretical foundation, our study delves into how the buoyancy and rhythmic breathing associated with swimming in water promote emotional relaxation, particularly contributing to significant improvements in emotional relaxation and stability. This refinement of environmental variables validates swimming's unique value in emotional intervention. Additionally, most existing research focuses on single psychological health indicators, such as changes in anxiety or depression (Smith and Merwin, 2021; Shen, 2022). In contrast, this study utilized a multi-dimensional psychological health assessment framework, covering three core dimensions—emotional state, social adaptation, and academic stress—along with nine specific indicators. This approach allows for a more comprehensive reflection of the multi-level impact of swimming on mental health. Additionally, current university mental health fitness programs primarily focus on land-based activities such as yoga, meditation, and running. While these activities can promote mental wellbeing to some extent, they have certain limitations, including high physical strain, limited effectiveness in emotional regulation, and insufficient social support (Martin et al., 2024; Al-Wardat et al., 2024). In contrast, swimming, as a full-body exercise intervention, offers more adaptable physiological regulation, emotional relief, and social support. The buoyancy of water reduces the risk of exercise-related injuries, making it accessible to a broader student population. The rhythmic breathing patterns in swimming help regulate the autonomic nervous system, effectively reducing anxiety levels, while the immersive water environment minimizes external distractions, enhancing psychological relaxation (Zhao, 2024). Swimming often involves teamwork and social interaction, fostering a greater sense of belonging and psychological support compared to individualized activities such as running or meditation. Therefore, this study proposes swimming as an effective complementary intervention that not only addresses the shortcomings of traditional university fitness programs but also provides a more physiologically adaptive, emotionally regulating, and socially engaging exercise option for university mental health strategies.

The theoretical contribution of this study primarily lies in highlighting the unique value of swimming as a multidimensional intervention approach. Compared to traditional psychological interventions, swimming not only promotes mental health through physical activity but also leverages the unique properties of the aquatic environment—such as facilitating muscle relaxation and enhancing blood circulation—to help students alleviate stress. Additionally, the teamwork and interaction involved in swimming training provide students with psychological support, strengthening their social confidence and further enhancing their adaptability and emotional regulation abilities. The combined effects of these mechanisms establish swimming as an intervention method that offers both physiological benefits and psychological advantages.

In practice, this study suggests that universities incorporate swimming and other exercise-based interventions into their mental health education systems, utilizing physical activity as a key strategy for promoting psychological wellbeing. Swimming provides a deeper level of relaxation through water buoyancy, rhythmic breathing, and mind-body coordination, helping to alleviate anxiety, depression, and academic stress. Therefore, universities can systematically integrate swimming into their mental health strategies through the following measures. First, incorporating swimming into mental health curricula and intervention programs. Universities can introduce swimming modules within mental health education courses, offering multi-level instruction from basic skills to advanced training to ensure accessibility for students of all fitness levels. University mental health centers or wellness programs can offer swimming-based psychological intervention courses, such as “Swimming for Emotional Regulation” or “Stress-Relief Swimming Sessions,” using structured training to help students manage psychological stress and enhance emotional stability. Second, establishing incentive mechanisms to encourage student participation in swimming programs. At the policy level, universities can integrate swimming into academic credit evaluation systems, making it part of the curriculum to increase student participation. Additionally, institutions can implement exercise incentive programs by introducing “Mental Health Fitness Scholarships” or “Sports Exercise Reward Programs” to encourage consistent engagement in swimming. To lower financial barriers, universities can provide free access to swimming facilities during designated hours or offer specialized financial subsidies to ensure all students have the opportunity to participate in swimming activities. Finally, aligning swimming interventions with university-wide mental health strategies to establish a dual “mental health + physical exercise” intervention system. University counseling centers can collaborate with sports departments to integrate swimming into mental health intervention plans, offering personalized exercise guidance for students experiencing anxiety, depression, or academic stress, with regular evaluations of its effectiveness. Schools can establish “Swimming Support Groups” where students train under the supervision of professional coaches and mental health counselors, fostering social support and reducing psychological distress. Additionally, universities can explore the integration of swimming with Cognitive Behavioral Therapy (CBT) by incorporating mindfulness meditation or self-affirmation exercises before and after swimming sessions to enhance emotional regulation. Universities may also equip swimming facilities with mental health resources, such as designated mental health information corners or wellness workshops, transforming them into multifunctional spaces for mental health promotion.

However, this study has some limitations. The sample size was relatively small, with only 44 students in the experimental and control groups, which limits the generalizability of the findings. Additionally, the research design was longitudinal, with a 15-week intervention period. While the short-term effects of swimming on mental health were observed, the study did not explore its long-term impact. The relatively short intervention period may lead to an overestimation or underestimation of the intervention effects, as the sustained impact of swimming on mental health could not be fully assessed. Future research should aim to include larger sample sizes and extend the duration of the intervention to better understand the lasting effects of swimming on psychological health.

## 6 Conclusion

This study involved 44 university students, divided into an experimental group and a control group, with the experimental group undergoing 15 weeks of swimming training to investigate the impact of swimming intervention on mental health. The specific research findings are as follows:

(1) Prior to the intervention, no significant differences were observed between the experimental and control groups in terms of baseline mental health indicators, including emotional state, social adaptability, and academic stress. Specifically, the average score for emotional relaxation in the experimental group was 3.26 ± 0.81, compared to 3.15 ± 0.81 in the control group, with a *P*-value of 0.67, indicating negligible differences in daily emotional relaxation between the two groups. Similarly, for positive emotional experiences, the experimental group scored 3.75 ± 0.74, while the control group scored 3.63 ± 0.72 (*P* = 0.69). In emotional stability, the experimental group scored 3.52 ± 0.91, and the control group scored 3.48 ± 0.92 (*P* = 0.72), showing no significant differences in pre-intervention emotional stability. In terms of social adaptability, the scores for interpersonal harmony, social support, and communication confidence were also very similar between the two groups, suggesting equivalent baseline conditions. Furthermore, there were no significant differences in academic stress across various dimensions, ensuring the fairness of the experimental design and the reliability of the subsequent results.
(2) After 15 weeks of swimming intervention, the experimental group showed significant improvements in multiple aspects of mental health. In terms of emotional state, the emotional relaxation score in the experimental group increased from 3.26 ± 0.81 before the intervention to 3.82 ± 0.75 after the intervention, while the control group remained at 3.25 ± 0.61 (*P* = 0.02). Positive emotional experience scores in the experimental group increased from 3.75 ± 0.74 to 4.07 ± 0.71, while the control group scored 3.68 ± 0.74 (*P* = 0.01). Emotional stability in the experimental group improved to 3.85 ± 0.78, compared to 3.52 ± 0.80 in the control group (*P* = 0.01), indicating that swimming effectively promoted emotional stability and enhanced positive emotional experiences. Regarding social adaptability, the experimental group showed significant improvements in interpersonal harmony and social support, with scores rising from 3.92 ± 0.77 and 4.03 ± 0.82 to 4.14 ± 0.73 and 4.21 ± 0.75, respectively. In contrast, the control group had scores of 3.83 ± 0.76 and 3.90 ± 0.77 (*P* < 0.05). Communication confidence also significantly increased to 4.05 ± 0.78 in the experimental group, compared to 3.78 ± 0.79 in the control group (*P* = 0.04). These results suggest that swimming provided a positive social environment for university students, enhancing their social confidence and sense of social support. In terms of academic stress management, the experimental group's academic composure increased from 2.92 ± 0.81 to 3.57 ± 0.82, while the control group's score was 3.05 ± 0.83 (*P* = 0.02). The experimental group's sense of academic achievement rose to 3.84 ± 0.75, compared to 3.48 ± 0.77 in the control group (*P* = 0.04). Additionally, goal management ability significantly improved in the experimental group, with a score of 3.73 ± 0.78, compared to 3.11 ± 0.80 in the control group (*P* = 0.04), indicating that swimming intervention helped enhance self-discipline and goal management abilities in students.

In conclusion, this study rigorously validated the positive impact of swimming intervention on mental health, particularly in enhancing emotional stability, social adaptability, and academic stress management. Future research could explore the following aspects: first, increasing sample sizes and including participants from different age groups, genders, and social backgrounds (such as high school students, graduate students, or even non-college individuals) to improve the generalizability and applicability of the findings. This would help further understand the impact of swimming intervention on mental health across different populations. Second, future studies could design experiments with a longer duration (6 months to a year) to systematically examine the long-term stability and sustainability of swimming's effects, thus providing a more accurate assessment of its long-term influence on mental health.

## Data Availability

The original contributions presented in the study are included in the article/supplementary material, further inquiries can be directed to the corresponding author.

## Appendix

Mental Health Assessment Questionnaire Instructions:

This questionnaire consists of 27 items covering three dimensions: emotional state, social adaptation, and academic stress. Please rate each item based on your actual experience using the following scale:

1 = Completely disagree

2 = Somewhat disagree

3 = Neutral

4 = Somewhat agree

5 = Completely agree

Section 1: Emotional State

- I feel emotionally calm in my daily life.
- I rarely feel anxious or nervous.
- I can effectively cope with daily stress and remain calm.
- I often feel happy and content.
- I feel confident about my life.
- I can experience positive emotions such as happiness and joy.
- My emotions are not easily influenced by external factors.
- I can control my emotions well and avoid mood swings.
- When facing setbacks, I am able to maintain emotional stability.

Section 2: Social Adaptation

- I have harmonious relationships with classmates, friends, and family.
- I feel comfortable and relaxed when interacting with others.
- I can easily establish good relationships with others.
- I feel that people around me support and help me.
- When I need help, I can rely on my family, friends, or others.
- I receive sufficient emotional support and social care.
- I am confident in expressing my views in social settings.
- I do not feel nervous or uneasy when communicating with others.
- I believe my communication skills meet daily needs.

Section 3: Academic Stress

- I can remain calm and composed when facing academic tasks.
- I believe I can manage my time effectively to complete academic tasks.
- I can stay calm and confident when dealing with academic stress.
- I am satisfied with my academic performance.
- I feel that my efforts in academics are being rewarded.
- My academic progress makes me feel proud and accomplished.
- I can clearly set academic goals and formulate plans to achieve them.
- I have the self-discipline to execute my plans and achieve my goals.
- I can effectively manage my time and tasks in the learning process.

Thank you for your participation!
